# Erythropoietin Mimetic Peptide (pHBSP) Corrects Endothelial Dysfunction in a Rat Model of Preeclampsia

**DOI:** 10.3390/ijms21186759

**Published:** 2020-09-15

**Authors:** Mikhail Korokin, Vladimir Gureev, Oleg Gudyrev, Ivan Golubev, Liliya Korokina, Anna Peresypkina, Tatiana Pokrovskaia, Galina Lazareva, Vladislav Soldatov, Mariya Zatolokina, Anna Pobeda, Elena Avdeeva, Evgeniya Beskhmelnitsyna, Tatyana Denisyuk, Natalia Avdeeva, Olga Bushueva, Mikhail Pokrovskii

**Affiliations:** 1 Department of Pharmacology and Clinical Pharmacology, Institute of medicine, Belgorod State National Research University, 308015 Belgorod, Russia; produmen@yandex.ru (V.G.); gudyrev@mail.ru (O.G.); golubevvano@yandex.ru (I.G.); korokina@mail.ru (L.K.); peresypkina_a@bsu.edu.ru (A.P.); pokrovskaia@bsu.edu.ru (T.P.); zinkfingers@gmail.com (V.S.); pobeda@bsu.edu.ru (A.P.); evgeny_b89@mail.ru (E.B.); 7400468@mail.ru (N.A.); pokrovskii@bsu.edu.ru (M.P.); 2 Department of Obstetrics and Gynecology FPE, Kursk State Medical University, 305000 Kursk, Russia; lazarevaga@kursksmu.net; 3 Institute of Gene Biology of the Russian Academy of Sciences, Core Facility “Genome editing”, 119334 Moscow, Russia; 4 Department of Histology, Embryology, Cytology, Kursk State Medical University, 305000 Kursk, Russia; marika1212@mail.ru; 5 Department of Normal Physiology, Kursk State Medical University, 305000 Kursk, Russia; avdeyeva_ev@mail.ru; 6 Department of Pharmacology, Kursk State Medical University, 305000 Kursk, Russia; denitatyana@yandex.ru; 7 Research Institute for Genetic and Molecular Epidemiology, Kursk State Medical University, 305000 Kursk, Russia; olga.bushueva@inbox.ru

**Keywords:** endothelial dysfunction, preeclampsia, erythropoietin-derived peptide, pHBSP, placenta, Wistar rats

## Abstract

Preeclampsia is a severe disease of late pregnancy. Etiological factors and a pathogenetic pattern of events still require significant clarification, but it is now recognized that a large role is played by placentation disorders and emerging endothelial dysfunction. The administration of short-chain peptides mimicking the spatial structure of the B erythropoietin chain may become one of the directions of searching for new drugs for preeclampsia prevention and therapy. Simulation of ADMA-like preeclampsia in Wistar rats was performed by the administration of a non-selective NOS blocker L-NAME from the 14th to 20th day of pregnancy. The administration of the pHBSP at the doses of 10 µg/kg and 250 µg/kg corrected the established morphofunctional disorders. The greatest effect was observed at a dose of 250 µg/kg. There was a decrease in systolic and diastolic blood pressure by 31.2 and 32.8%, respectively (*p* < 0.0001), a decrease in the coefficient of endothelial dysfunction by 48.6% (*p* = 0.0006), placental microcirculation increased by 82.8% (*p* < 0.0001), the NOx concentration was increased by 42,6% (*p* = 0.0003), the greater omentum edema decreased by 11.7% (*p* = 0.0005) and proteinuria decreased by 76.1% (*p* < 0.0002). In addition, there was an improvement in the morphological pattern of the fetoplacental complex and the ratio of BAX to Bcl-2 expression which characterizes the apoptotic orientation of the cells.

## 1. Introduction

Preeclampsia is a serious complication of late pregnancy [1,2]. Every year, 50,000 women die from preeclampsia and its complications [3]. According to various authors, preeclampsia develops with a frequency of 2% to 10% of all pregnancies [4,5,6]. In 0.03–0.055%, it passes into eclampsia [7]. Preeclampsia takes a leading place after bleeding in the structure of maternal and child morbidity and mortality in developed and developing countries [8,9]. In many developing countries, preeclampsia accounts for 40% of maternal deaths [7]. In developed countries, it accounts for about 16–18% of maternal mortality and up to 40% of fetal and newborn deaths [10]. In women with preeclampsia, the rate of complications can reach 22% [4]. Preeclampsia is the main cause of preterm birth, intrauterine growth restriction, and perinatal mortality [11]. Thus, in healthy women, the probability of preterm birth is 7.2%, in pregnant women with hypertension, it increases to 12.5%, in women with preeclampsia, it reaches 39.2% [4].

The exact pathogenesis of this disease is not yet known and will be the subject of debate and scientific research for a long time. However, conducted analysis already allows us to speak about certain patterns in the occurrence of preeclampsia [8]. Since this disease occurs at the placenta formation and is eliminated when it is separated, it is logical to assume that the pathogenesis, or at least some of its events, should be associated with this organ [11]. The main pathogenetic events can be divided into two stages. Most authors believe that the triggering point of this pathology is a violation of placentation due to inadequate invasion of the trophoblast into the spiral arteries of the uterus [10]. This leads to impaired microcirculation in the placenta and trophoblast ischemia [6,10]. In response, a large number of humoral factors are released that have a pronounced effect on the vascular tone. Therefore, one of the ways of early preeclampsia diagnosis is to determine these factors: PlGF, sFlt-1, sEng, PAPP-A, and others [6,10].

The next stage in the pathogenesis of preeclampsia is a systemic violation of endothelial tone, leading to multiple organ failure. It should be noted that factors that increase the risk of preeclampsia developing are a history of preeclampsia, young age of a mother, first pregnancy, diabetes, hypertension, antiphospholipid syndrome, thrombophilia, autoimmune disease, kidney failure, infertility, remarriage, abortions in history, multiple pregnancies, fetal gender, obesity, etc. [3,6].

In the morphological study of the placenta of women who came through preeclampsia, researchers note violations of its formation. This is manifested in not fully ingrowth of the chorion villi into the mother’s spiral arteries. In this case, the spiral arteries retain their layers up to the muscular one, which leads to trophoblast ischemia [10]. The response to ischemia is the release of a large number of humoral factors, the action of which ultimately leads to the development of endothelial dysfunction [6,10]. In this regard, we suppose that anti-ischemic drugs can have a pronounced endothelioprotective activity. However, the information that preeclampsia develops more often in women with endothelial-associated diseases, as well as the variety of clinical variants of its manifestation, does not give an accurate answer to the question: “what is primary—placental ischemia or endothelial dysfunction”. It is obvious that endothelial dysfunction and placental ischemia are interconnected components of the preeclampsia pathogenesis.

Despite all the achievements of modern medicine, the only guaranteed treatment for preeclampsia is premature delivery, regardless of the duration of pregnancy [11,12]. Recently, there is a fraught debate about the introduction of drugs that improve endothelial function in therapy and prevention. The results in this area are still low-key, and the evidence base is still in the process of creation, but its prospects are obvious [13,14]. Despite this, targeted treatment reducing ischemic events in preeclampsia is not carried out (with the exception of rheolytics) [12].

We suppose that one of the ways of endothelial damage prevention is the administration of molecules with universal cytoprotective activity [15]. One of these molecules is the endogenous glycoprotein, erythropoietin (Epo). Epo is the main peptide that regulates erythropoiesis caused by hypoxia. However, Epo has other several important functions that contribute to successful placentation and vascular adaptation to pregnancy [16]. Epo performs non-hematopoietic functions, influencing the processes of apoptosis and proliferation of Epo-sensitive tissue cells [17,18]. Epo expression in the placenta probably demonstrates its paracrine role in the processes of survival, proliferation, and differentiation of placental trophoblast cells [19]. There is evidence that increased levels of erythropoietin lead to inhibition of the contractile activity of placental vessels, so an increase in Epo leads to an improvement in placental perfusion [20]. At the same time, recombinant erythropoietin used in doses that enhance erythropoiesis can lead to the development of preeclampsia [21]. This is one of the main limitations of using Epo during pregnancy.

In 2004, Michael Brines et al. proved that the non-hematopoietic effects of Epo are realized through the heterodimeric EPOR/CD131 complex. In 2008, the same authors presented generalized results of studying the cytoprotective activity of an 11-amino acid peptide based on the a-helix of B erythropoietin, which mimicking the spatial part of the molecule binding with the heterodimeric EPOR/CD131 receptor, but does not interact with the homodimeric EPOR/EPOR receptor. This peptide (pHBSP, cibenitide, PubChem CID: 91810664) demonstrated the ability to significantly improve the morphofunctional state of tissues in diabetic retinal edema, renal ischemia-reperfusion, and significantly improve cognitive functions in the galantamine-induced amnesia model, but the absence of any effect on erythropoiesis [22,23].

These facts served as the basis for this study.

## 2. Results

### 2.1. Results of Function Tests 

**Figure 1:**
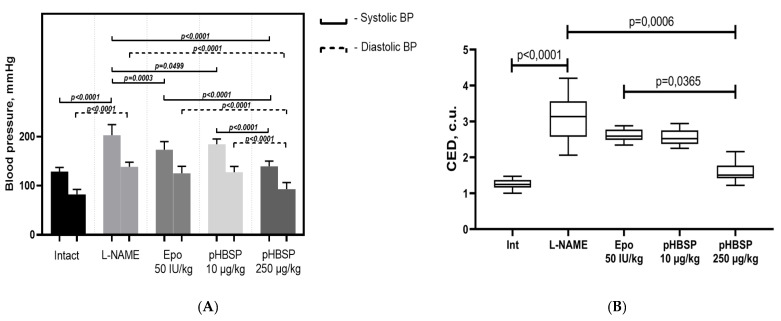
The effect of pHBSP on the blood pressure (**A**) and the coefficient of endothelial dysfunction (**B**) in animals with ADMA-like preeclampsia. Note: SBP, DBP – *n* = 10 animals in each group, systolic and diastolic blood pressure (mmHg); CED-coefficient of endothelial dysfunction (relative units, R.U.).

After simulation of endothelial dysfunction by the administration of N-nitro-L-arginine-methyl ether (L-NAME) in pregnant rats, systolic and diastolic blood pressure increased significantly (*p* < 0.0001) by 57.5 and 68.4% relative to intact animals, respectively. Administration of Epo 50 IU/kg, 11-amino acid peptide pHBSP (amino acid sequence QEQLERALNSS) at the doses of 10 µg/kg and 250 µg/kg to animals with experimental preeclampsia resulted in a decrease in systolic blood pressure by 14.6% (*p* = 0.0003), 9% (*p* = 0.0499) and 31.2% (*p* < 0.0001), and diastolic blood pressure by 9.5%, 7.9% and 32.8% (*p* < 0.0001), respectively, but the target level was not reached. (Figure 1A). Simulated ADMA (asymmetrical dimethylarginine)-like preeclampsia led to the development of endothelial dysfunction and violation of regulatory mechanisms of vascular tone, as evidenced by an increase in the coefficient of endothelial dysfunction (CED) by 148% (*p* < 0.0001) (Figure 1B). The administration of Epo 50 IU/kg, pHBSP for 10 days at the dosages of 10 µg/kg and 250 µg/kg in pregnant animals with ADMA-like preeclampsia led to a decrease in CED by 16.1%, 17.7%, and 48.6% (*p* = 0.0006), indicating improved endothelial function. When using pHBSP for 10 days kg at a dose of 250 μg/kg, the coefficient of endothelial dysfunction was statistically significantly lower than when using Epo (*p* = 0.0365).

**Figure 2:**
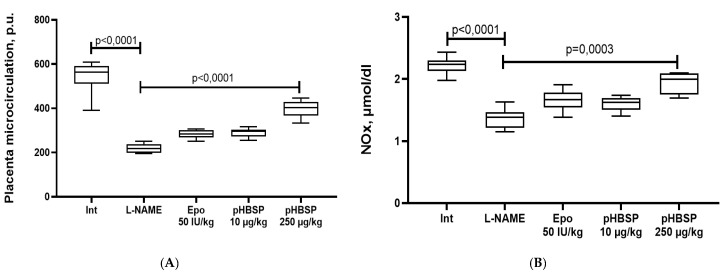
The effect of pHBSP on placental microcirculation (**A**) and the concentration of final stable nitric oxide metabolites (NOx) (**B**) in animals with ADMA-like preeclampsia (*n* = 10 animals in each group).

In animals with ADMA-like preeclampsia, a decrease in microcirculation was observed by 59.8% (*p* < 0.0001). The administration of pHBSP to animals with experimental preeclampsia at the dose 250 µg/kg resulted in an increase in the level of placental microcirculation by 82.8% (*p* < 0.0001); however, the target level of this indicator in the group of intact animals was not reached (Figure 2A).

A study of the NO-synthesizing function of the endothelium was performed by determining the concentration of final stable nitric oxide metabolites NOx. The administration of L-NAME to females from 14 to 21 days of pregnancy resulted in a decrease in the NOx concentration in blood plasma by 38.7% (*p* < 0.0001) relative to intact animals. Administration of pHBSP at the dose 250 µg/kg resulted in a significant increase (*p* = 0.0003) in the concentration of nitrite ions (NOx) in the blood plasma in animals with ADMA-like preeclampsia by 42.6% relative to an L-NAME group (Figure 2B).

**Figure 3:**
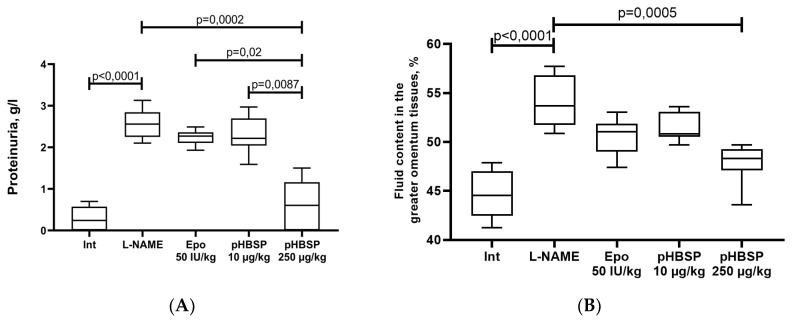
The effect of pHBSP on the proteinuria (**A**) and the fluid content in the greater omentum tissues (**B**) in animals with ADMA-like preeclampsia (*n* = 10 animals in each group).

Simulated ADMA-like preeclampsia caused an increase in proteinuria by 8.9 times (*p* < 0.0001). Administration of pHBSP at the dose 250 µg/kg from 10 to 20 days in pregnant animals with ADMA-like preeclampsia significantly reduced the urine protein by 76.1% relative to the L-NAME group, but these values did not reach the target values in intact females. The effect of the dose of 250 µg/kg significantly exceeded the effect of administration of Epo50 IU/kg (*p* = 0.02) and pHBSP 10 µg/kg ((*p* = 0.0087) Figure 3A).

In pregnant rats with ADMA-like preeclampsia, an increased fluid content in the greater omentum tissue by 21% (*p* < 0.0001) was observed (Figure 3B). The administration of Epo and pHBSP from 10 to 20 days of pregnancy at the dose of 10 µg/kg does not significantly reduce the fluid content in the greater omentum tissue. In addition, in the group of animals that received pHBSP 250 µg/kg, a significant decrease in the studied fluid content in the greater omentum tissue by 11.7% (*p* = 0.0005) was found (Figure 3B).

The results of the functional studies showed a pronounced dose-dependent effect of the studied peptide. It is noteworthy that the observed effects of the pHBSP at the dose of 10 µg/kg are comparable to the effects of the comparison drug—recombinant erythropoietin (50 IU/kg), and the effectiveness of pHBSP at the dose of 250 µg/kg significantly exceeded the effect of Epo.

**Figure 4:**
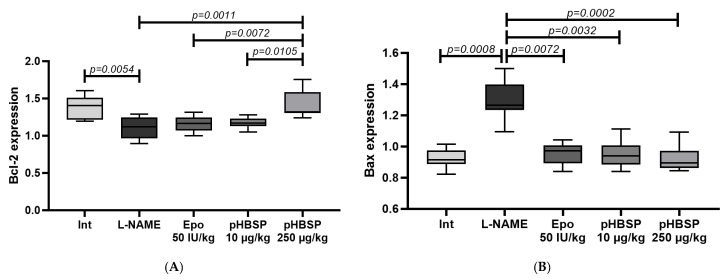
The level of Bax (**A**) and Bcl-2 (**B**) expression in animals with ADMA-like preeclampsia (*n* = 10 animals in each group).

qPCR showed that the level of Bcl-2 mRNA significantly decreased in the group administered with L-NAME for seven days relative to the intact animals (*p* = 0.0054). Ten-day therapy with Epo and pHBSP 10 µg/kg did not restore the expression of this anti-apoptotic factor. The best result was obtained in the group of rats treated with pHBSP at the dose of 250 µg/kg. The expression of Bcl-2 in this group turned out to be statistically significantly higher than in the L-NAME group, and groups of animals that received Epo and pHBSP 10 µg/kg (Figure 4A).

Expression of the pro-apoptotic factor Bax, in contrast, was significantly increased in the control group, and both drugs also showed a great effect on reducing Bax mRNA (Figure 4B). The ratio of Bax to Bcl-2 expression which characterizes the apoptotic orientation of the cells is presented in Table 1.

### 2.2. Results of Histological Examination 

**Figure 5:**
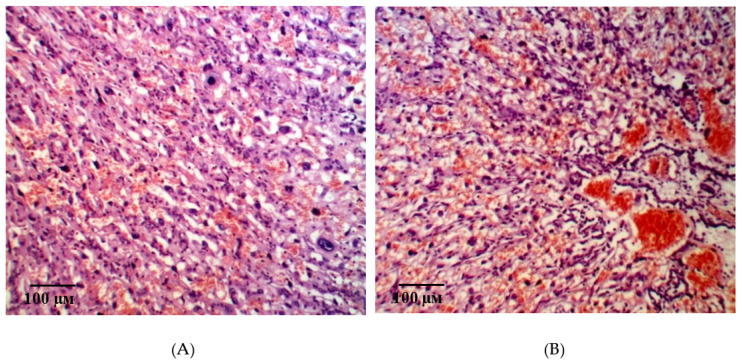
Microphotographs of the fetal placenta structures in intact animals on the 21st day of gestation. Note: a large number of structurally formed cotyledons formed by the stem villi (**A**) are in the field of view. The field of view is dominated by terminal villi, which have a small diameter and centrally located sinusoidal vessels. The vessels have a relatively small lumen, but they occupy almost the entire area of the villi (**B**). Hematoxylin and eosin staining. Magnification: 200×.

Two morphologically different parts of the placenta, fetal and maternal, were well visualized in histological examination of the placental microsections in intact animals on the 21st day of gestation. The fetal part consisted of the amniotic sac, connective tissue of the mucous type, as well as the chorial plate with derived villi is 3–3.5 times thicker than the maternal part. A large number of structurally formed cotyledons are in the field of view, the main part of which is made up of a stem and intermediate villi (Figure 5A). The field of view is dominated by terminal villi, which have a small diameter and centrally located sinusoidal vessels. The vessels have a relatively small lumen, but they occupy almost the entire area of the villi (Figure 5B).

**Figure 6:**
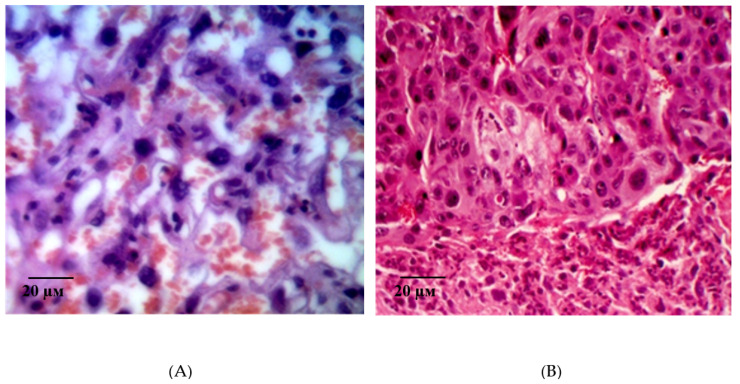
Microphotographs of the fetal placenta structures in intact animals on the 21st day of gestation. Note: The epithelium of the terminal villi is represented by syncytiotrophoblast. The microvasculature is represented by intermediate and sinusoid capillaries (**A**). A large number of enlarged decidual cells with pale cytoplasm and large trophoblast cells are arranged in several layers (**B**). Hematoxylin and eosin staining. Magnification: 400×.

The terminal villi epithelium is represented by a syncytiotrophoblast. Zones with nuclei and a homogeneous eosinophilic matrix are clearly defined visually. These structures of the epithelium and adjacent to the stroma and the capillary vessels wall form syncytial capillary platelets. Syncytial knots with hyperplastic dark basophilic nuclei are nearby. The microvasculature is represented by capillaries of intermediate and sinusoid types (Figure 6A). It is interesting to note that, on the side of the myometrium, branching villi are more organized and structured.

The second morphological part of the placenta, maternal, is well defined by the presence of decidua basalis, containing pale enlarged cells. In the thickness of the maternal placenta, lacunae filled with maternal blood and connective tissue trabecules between them are determined. The basal plate, located under the chorion villi, has a large number of decidual cells. The cells are enlarged, with pale cytoplasm and an oval nucleus, arranged in groups diffusely over the entire area (Figure 6B). Between them, blood lacunae filled with the mother’s blood are visualized. The field of view is dominated mainly by large, oval cells that form multi-layer sheets. Small decidual cells are isolated, their cytoplasm is dark basophilic, and the nucleus is dominated by heterochromatin. Small clusters of trophoblast cells, mostly enlarged, are located on the border between the fetal and maternal parts in several layers.

**Figure 7:**
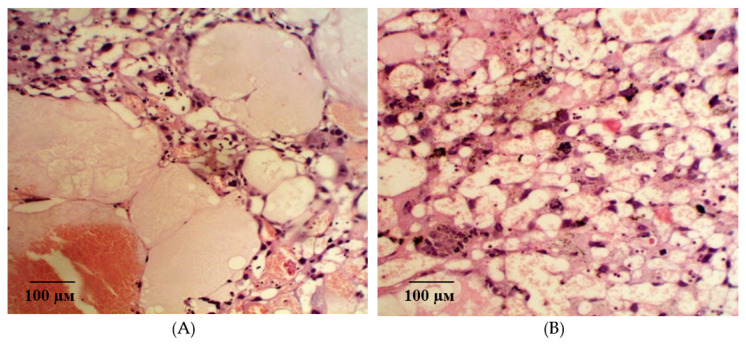
Microphotographs of the fetal placenta structures in control animals on the 21st day of gestation in simulated ADMA-like preeclampsia. Note: the area of the interstitial space is smaller compared to intact animals (**A**). Interstitial edema of small vessels and stroma of the villi. Local fibrin deposition. Hofbauer cells (**B**) are well visualized. Hematoxylin and eosin staining. Magnification: 200× (**A**,**B**).

Histological examination of the placental microsections in the control group in animals with simulated preeclampsia on the 21st day of gestation revealed the predominance of intermediate villi. They had a larger diameter and consisted of a well-developed stroma and centrally located blood vessels. Signs of interstitial inflammatory edema of the connective tissue stroma of the stem anchoring and terminal villi were visualized, and, as a result, their size increased and the spaces between them decreased (Figure 7A). Signs of edema are also found in the walls of small vessels. Focal hemorrhages and fibrin deposition were observed. Hofbauer cells were well visualized (Figure 7B).

**Figure 8:**
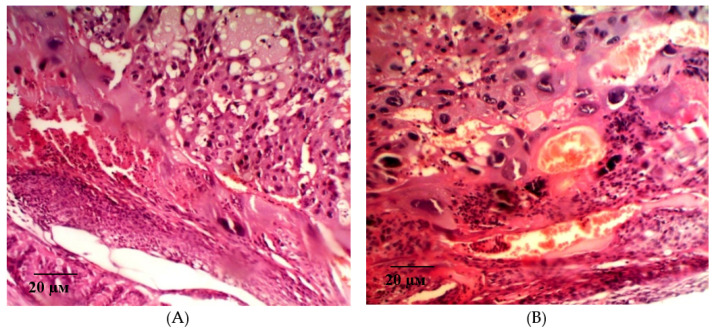
Microphotographs of the maternal placenta on the 21st day of gestation in control animals with ADMA-like preeclampsia. Note: Impaired structure of morphological elements of the placenta. Optically empty decidual cells (**A**). Extensive fields of necrosis and fibrin deposits (**B**). Hematoxylin and eosin staining. Magnification: 400× (**A**,**B**).

It should be noted that, in the microsections in the anchoring and terminal villi, the phenomena of angiomatosis, as well as sclerosis of their own blood vessels and the stroma of the villi were observed. The consequence of this process is a violation of histoarchitectonics and expansion of the interstitial space. In addition, in some villi, vascular thrombosis was detected, and in some villi, hemorrhages were localized both in the stroma and in the interstitial space. Deposits of fibrin filaments and elements of desquamated chorial epithelium were observed between the villi. The absence (or in a few cases) of syncytial capillary membranes and syncytial knots should be noted. This reaction is compensatory and adaptive in nature, aimed at eliminating violations and resuscitation the volume of microcirculation. It should be noted that, in the giant cell trophoblast, namely, in the visualized decidual membrane of the maternal placenta, inflammatory and dystrophic changes are pronounced. Optically empty decidual cells are determined against the background of cells with absolutely integrity structures (Figure 8A). The structure of the morphological elements is broken. Extensive focal fields of necrosis and fibrin deposits are in the field of view (Figure 8B). There is a pronounced deformation of decidual cells.

On the part of the chorial and basal plates, as well as the stroma of the terminal villi, there is a moderate focal lymphoplasmacytic infiltration with a small amount of segmentonuclear leukocytes.

**Figure 9:**
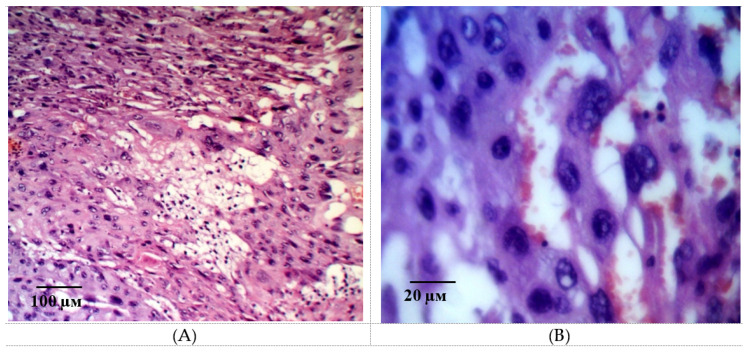
Microphotographs of the placenta on the 21st day of gestation in animals with ADMA-like preeclampsia when pHBSP was administered at the dose of 10 µg/kg. Note: Hematoxylin and eosin staining. Magnification: 200× (**A**) and 400× (**B**).

In the group of rats with ADMA-like preeclampsia, after the administration of pHBSP at the dose of 10 µg/kg, some degenerative-dystrophic changes in the placenta continue to persist against the background of a slight positive dynamics. In the fetal placenta, signs of edema are expressed in the stroma of the villi. Blood vessels located here are spasmodic, narrow, and, in most cases, empty or with signs of thrombosis. The terminal and intermediate villi prevailing in the field of view are surrounded by a layer of syncytiotrophoblast, which is destroyed in some areas and substituted with oxyphilic fibrinoid. Insufficient mature of the placenta by this time should be noted, and syncytial knots are isolated in the field of view (Figure 9A). There is a thickening of the giant-cell trophoblast in the maternal part of the placenta. Decidual cells located in layers are with dystrophic changes. There are large local areas with optically empty decidual cells and single areas of necrosis in the thickness of the trophoblast (Figure 9B).

In the group of rats with ADMA-like preeclampsia, a moderate positive dynamic of morphological changes was observed when pHBSP was administered at the dose of 250 µg/kg. There is a decrease in inflammatory edema of the villi stroma, against the background of blood-filled vessels. The field of view is dominated by terminal and intermediate villi, but there is a significant decrease in the number and density of syncytial knots, indicating the degree of the placenta maturity. Foci of fibrinoid covering the villi in the absence of syncytiotrophoblast are moderate.

**Figure 10:**
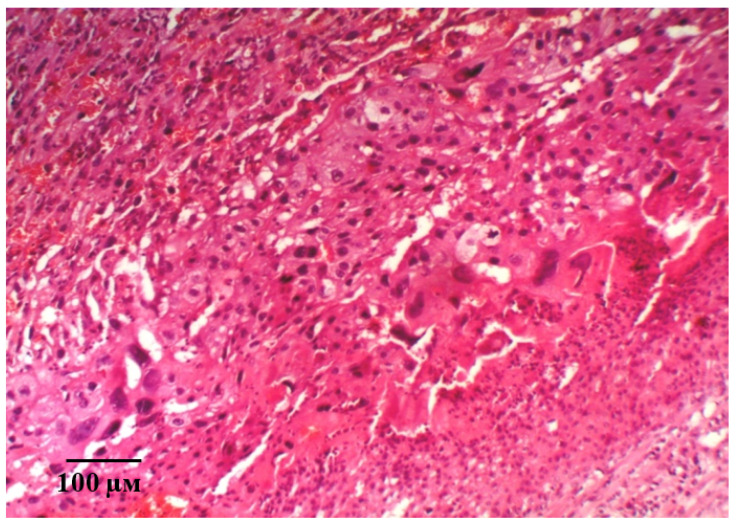
Microphotograph of the placenta on the 21st day of gestation in animals with ADMA-like preeclampsia when pHBSP was administered at the dose of 250 µg/kg. Hematoxylin and eosin staining. Magnification: 200×.

A significant number of decidual cells with dystrophic changes are detected in the giant cell trophoblast. Large foci of round-cell infiltration with merger trends are visualized on the trophoblast periphery. Small local areas of necrotically altered tissue are determined in the maternal placenta (Figure 10).

In the thickness of these layers of decidual cells, areas formed by optically empty decidual cells with dark basophilic nuclei are visualized, in immediate proximity to which merged symplastic structures—Hofbauer cells are determined.

**Figure 11:**
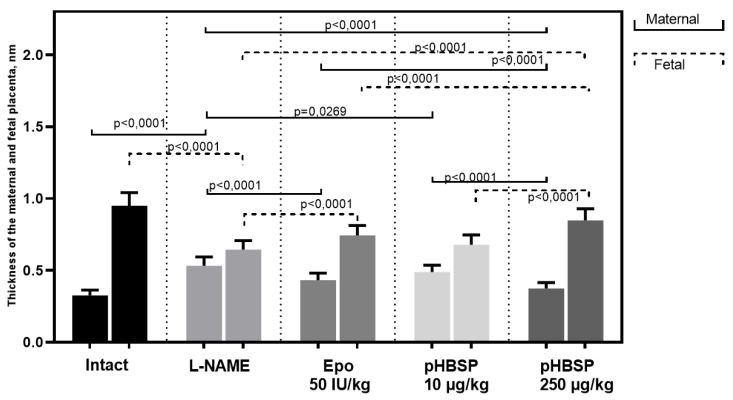
The effect of pHBSP on the thickness of the maternal and fetal placenta in animals with experimental preeclampsia (*n* = 10 animals in each group).

**Figure 12:**
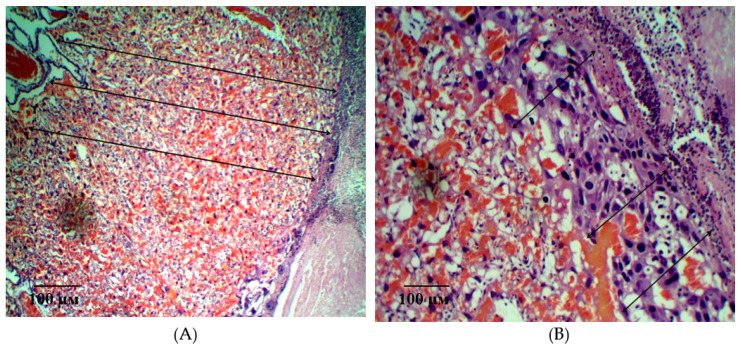
Measurement of the thickness of the fetal (**A**) and maternal (**B**) parts of the placenta on the 21st day of gestation in animals with ADMA-like preeclampsia when pHBSP was administered at the dose of 250 µg/kg. Note: Hematoxylin and eosin staining. Magnification: 200×.

The described changes were accompanied by positive dynamics in the size of the fetal and maternal parts of the placenta (Figure 11). An example of measuring the thickness of the maternal and fetal parts of the placenta is shown in Figure 12A,B. 

In addition, there was a partial restore of the cellular density of the placental tissue component of the maternal and fetal placenta and the diameter of the villi (Table 2).

**Figure 13:**
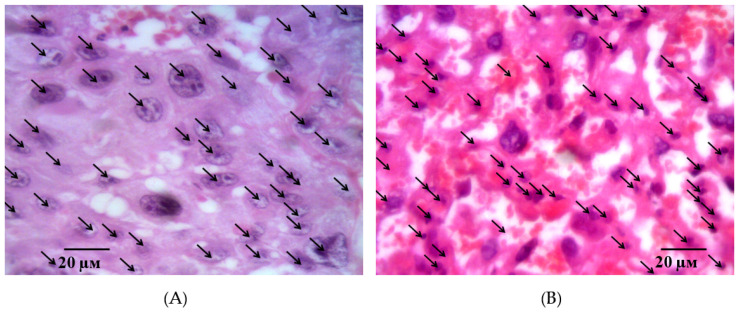
Measurement of the density of decidual cells (**A**, indicated by arrow) and density of cells of the fetal part of placenta (**B**, indicated by arrow) on the 21st day of gestation in animals with ADMA-like preeclampsia when pHBSP was administered at the dose of 250 µg/kg. Note: Hematoxylin and eosin staining. Magnification: 400×.

**Figure 14:**
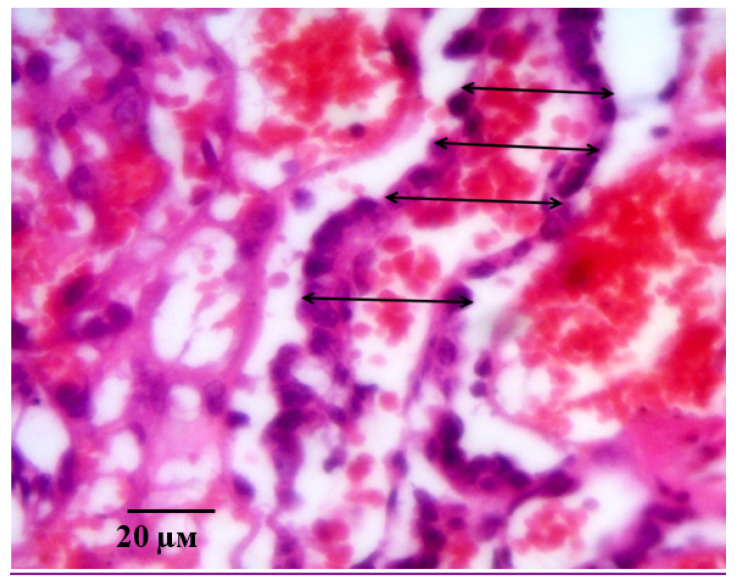
Measurement of the villi diameter (indicated by arrow) on the 21st day of gestation in animals with ADMA-like preeclampsia when pHBSP was administered at the dose of 250 µg/kg. Note: Hematoxylin and eosin staining. Magnification: 400×.

An example of measuring the density of decidual cells is shown in Figure 13A, measuring the density of cells of the fetal part of the placenta in Figure 13B. Figure 14 shows an example of a villi diameter measurement.

## 3. Discussion

The results of the present study confirm our hypothesis that drugs with cytoprotective and anti-ischemic effects can indirectly affect endothelial dysfunction. However, data that preeclampsia develops more often in women with diseases associated with endothelial dysfunction, as well as a variety of clinical variants of its manifestation, do not give an accurate answer to the question: “what is primary—placental ischemia or endothelial dysfunction?” However, it is obvious that endothelial dysfunction and placental ischemia are interrelated components of the pathogenesis of preeclampsia.

Based on the available literature data on the main pathogenetic points in the development of preeclampsia, we suppose that an effective direction in the prevention and treatment of this pathology could be the practical application of drugs with anti-ischemic, cytoprotective, and antihypoxic activity.

Erythropoietin is a glycoprotein with a molecular weight of 31 kDa. In the classical view, erythropoietin is a regulator of erythropoiesis and is produced in the kidneys in response to ischemia. In other tissues, the hyposialated form is produced, which plays a greater role in local regulatory processes aimed primarily at cytoprotection [23]. Previous studies have shown high protective activity of erythropoietin in various experimental pathologies and in many pathological conditions [24,25,26]. However, the use of erythropoietin is limited due to the side effects such as erythrocytosis, increased thrombosis, hypertension, growth progression, and the formation of new tumors [17,24,27].

Currently, systemic pleiotropic protective effects of Epo in conditions of its increased production have been identified. According to the literature, Epo increases the duration of the functioning of endothelial cells during oxygen deficiency, has an anti-apoptotic effect on endothelial cells, causes activation of endothelial NO-synthase, and prevents angiospasm, reduces NO-toxicity, and has a direct antioxidant effect.

“Non-classical” activity of erythropoietin allows us to consider it as a therapeutic agent for the prevention of conditions associated with necrosis, apoptosis, and inflammatory processes in tissues, in particular, in the treatment of preeclampsia.

Particular interest in erythropoietin is caused by studies showed that it is possible to separate the erythropoietic and non-erythropoietic effects of erythropoietin [25,28]. Homodimeric receptor (EPOR/EPOR) is on the megakaryocytes of the bone marrow [24,26]. The signaling pathway after its activation begins with the phosphorylation of JAK2 [29]. In other tissues, the heterodimeric receptor (EPOR-βCR) is located, or it is also called the innate repair receptor (IRR) [24,27,30,31]. The start of the signaling pathway after its activation also begins with the phosphorylation of JAK2; then, the activation of different signaling pathways leads to the implementation of non-erythropoietic effects depending on the tissues [30,31,32,33]. The discovery of the fact that the erythropoietic and tissue-protective properties of Epo are realized through two different receptor systems led to the opportunity for a fundamentally new direction in the search for new erythropoietin derivatives with cytoprotective and endothelioprotective activity.

Such derivatives can be considered asialo-erythropoietin and carbamylated darbepoetin. The lack of the effect of these derivatives on erythropoiesis is due to a short half-life or selective binding to EPOR-βCR. However, asialo-erythropoietin and carbamylated darbepoetin are plenty large molecules, which is not ideal for penetration of tissue barriers.

In this regard, the next stage in the development of drugs based on compounds that selectively activate the heterodimeric erythropoietin receptor was the creation of short-chain peptides mimicking the spatial structure of the B erythropoietin helix [25,34,35,36]. This derivative was an 11- amino acid peptide with the sequence Phe-Glu-Gln-Leu-Gly-Arg-Ala-Leu-Asn-Ser-Ser (pHBSP, ARA 290) [34,35,37].

In experimental studies, pHBSP has been shown to increase the resistance of the retina to ischemia [38], has an anti-inflammatory effect in simulated colitis in mice [39], accelerates reparative processes in diabetes [40], improves the results of pancreatic islet transplantation [41], and has cardioprotective and neuroprotective effects [25].

The main area of research on pHBSP in clinical practice was its use in patients with sarcoidosis. At the same time, it reduces fatigue [42] and has pronounced neuroprotection in this disease [31,39].

In the context of our study of the activity of pHBSP in experimental preeclampsia, it should be noted that the chosen model of preeclampsia has an ischemic component despite the fact that it is caused by a vasoactive ADMA-like substance. Apparently, placental vessels are most sensitive to L-NAME, which causes their spasm and subsequent ischemia up to necrotic alteration [43].

When modeling preeclampsia by the administration of L-NAME, we found pathological changes in the placenta formation. Incomplete germination of chorion villi into the coiled arteries of the uterus occurs. At the same time, the coiled arteries retain their layers up to the muscular one, which leads to trophoblast ischemia. The response to ischemia is the release of a large number of humoral factors, the action of which ultimately leads to the development of endothelial dysfunction. Our results conclusively prove the pronounced dose-dependent positive effects of pHBSP (laboratory code pHBSP) on the correction of morphofunctional disorders in animals with ADMA-like preeclampsia. The mechanism of protective action is the ability of pHBSP to selectively bind to the heterodimeric erythropoietin receptor. In this case, non-erythropoietic effects specifically attributed to recombinant erythropoietin are realized: anti-ischemic, anti-apoptotic, and anti-inflammatory [34,35,37]. This contributes to cytoprotective effects in the fetoplacental complex, reducing the formation of humoral factors that cause endothelial dysfunction and eNOS activation. The results obtained during histological examination also indicate that pHBSP is able to prevent inflammatory and ischemic changes in the placenta of the L-NAME-induced preeclampsia. This phenomenon is most likely related to both the anti-apoptotic activity of the compound and the intrinsic anti-inflammatory activity of erythropoietin-type molecules.

Apoptosis is known to play a key role in the physiology of the human placenta [44]. The Bcl-2 and Bax genes are the most important genes that control and regulate apoptosis. It was found that the Bcl-2 and Bax genes act in concert to maintain programmed cell death in the placenta [45]. Abnormal apoptotic activity in the placenta may be caused by a violation of the regulation of these genes. We have obtained evidence that Epo and pHBSP significantly inhibit L-NAME-induced increase in the expression of Pro-apoptotic factor Bcl-2. At the same time, an L-NAME-induced decrease in the expression of the pro-apoptotic factor Bax was found. When using a suberythropoietic dose of Epo, as well as pHBSP at a dose of 10 µg/kg, we did not establish a positive dynamics of Bax. A statistically significant increase in Bax expression in the group of animals receiving pHBSP at a dose of 250 µg/kg also led to a decrease in the ratio of Bax expression to Bcl-2 expression. Normalization of the ratio of Bax expression to Bcl-2 expression was also found in experimental groups receiving Epo and pHBSP at a dose of 10 µg/kg. This property is very significant for a potential drug for the treatment of endothelial-associated diseases.

The direction of further development of erythropoietin mimetic peptides may be the modification of helix B surface peptide pHBSP to change its pharmacokinetic and pharmacodynamic characteristics to obtain the necessary physiological effects. Taking into account the presence of prothrombotic properties of erythropoietin derivatives, we think that one of these directions is the modification of pHBSP by attaching peptide motifs with antiplatelet activity. As such motifs, we consider the tripeptides RGD (Arg-Gly-Asp) and KGD (Lys-Gly-Asp). It is known that these motifs have pronounced antiplatelet properties [46,47,48]. In parallel, the PGP Tripeptide (Pro-GlyPro) can be added to improve the pharmacokinetic characteristics. This amino acid sequence stabilizes the molecule in biological environments by inhibiting the activity of proteolytic enzymes [49]. Moreover, PGP can also block the angiotensin-converting enzyme [50], one of the most important pro-atherogenic factors that catalyze the formation of angiotensin II, increasing the risk of brain stroke [51]. Several studies have also identified antithrombotic and antiplatelet properties of PGP [52,53,54], which makes it even more promising to join pHBSP. The key question remains the nature of embedding RGD and PGP motifs in the base molecule.

## 4. Materials and Methods

### 4.1. Animals

The experiments were approved by the Belgorod State National Research University, Local Ethics Committee, Belgorod (Protocol #07/19 from 15.04.2019). Ethical principles of handling laboratory animals were observed in accordance with the European Convention for the Protection of Vertebrate Animals Used for Experimental and Other Scientific Purposes, CETS No. 123. The animals were housed in an animal facility with a 12-h day/12-h night cycle and provided a standard laboratory diet and water. The study was performed in 170 female Wistar rats weighting 250–300 g.

At the period of the study, the animals were healthy, with no changes in behavior, appetite, or sleep–wake schedule. For 18 h before the experiments, the animals were under the condition of complete food deprivation with free access to water.

To form groups of pregnant animals that were kept separately, males (2 animals) were introduced to females (3 animals) for a day. Then, the animals were separated and after 10 days, to diagnose pregnancy, under the drug-induced sleep (Zoletil ^®^ (Tiletamine+Zolazepam) 50 mg/kg of rat body weight, i.p.), the fact of pregnancy was determined by palpation of the anterior abdominal wall. For 12 h before the diagnosis of pregnancy, the animals were deprived of food. The animals with palpatory confirmed pregnancy were taken into the experiment.

### 4.2. Experimental Design

The following groups were included in the experiment (10 animals in each group):

- 1 group—intact (animals with physiological pregnancy);
- 2 group—control (simulation of ADMA-like preeclampsia in the experimental animals was performed by administration of a non-selective NOS blocker L-NAME 25 mg/kg/day subcutaneously from the 14th to 20th day of pregnancy);
- 3 group—L-NAME + recombinant erythropoietin at the dose of 50 IU/kg/day subcutaneously from the 10th to 20th day of pregnancy;
- 4 group—L-NAME + pHBSP at the dose of 10 µg/kg/day subcutaneously from the 10th to 20th day of pregnancy;
- 5 group—L-NAME + pHBSP at the dose of 250 µg/kg/day subcutaneously from the 10th to 20th day of pregnancy.

On the 21st day of gestation, the experimental animal was anesthetized by intraperitoneal injection of chloral hydrate at the dose of 150 mg/kg + Zoletil 50 mg/kg of body weight, after which functional tests were performed [55,56,57].

### 4.3. Assessment of Endothelial Dysfunction

On the 43rd day from the start of the experiment, in each animal under anesthesia (Zoletil^®^ (Tiletamine+Zolazepam) 50 mg/kg + Chloral hydrate 150 mg/kg), the left carotid artery was catheterized for intravascular measurement SBP and DBP using the MP150 data acquisition and analysis system (Biopac Systems, Inc., Goleta, CA, USA). In continuous blood pressure measurement mode, vascular tests of endothelium-dependent (acetylcholine 40 μg/kg) and endothelium-independent (sodium nitroprusside, 30 μg/kg) vasodilation were performed. Vasoactive agents were administered at 15 min intervals through a catheter inserted in the femoral vein. The level of endothelial dysfunction in the experimental animals and also the level of its correction by studied drugs were valued by CED expressed in relative units. 

This coefficient was calculated by the following formula: CED = SBPNP/SBPAH, where SBPNP is the area of the triangle above a BP recovery curve at a functional test with nitroprusside administration; SBPAH is the area of the triangle above a BP recovery curve at a functional test with acetylcholine administration. Points of a smaller side of this triangle are the points of BP before the test and the point of maximum reduction of a BP, and the bigger side is the time of BP restoration [58,59].

### 4.4. Registration of Placental Microcirculation

The state of placental microcirculation was assessed using Biopac systems equipment: MP100 polygraph with LDF100C laser Doppler flowmetry module and TSD144 invasive needle sensor (Biopac Systems, Inc., Goleta, CA, USA), which was placed directly on the projection of the placental disk, 1 mm to the center from the outer edge. Registration and processing of LDF results were performed using the AcqKnowledge program version 3.8.1; the values of microcirculation were expressed in perfusion units [55].

After functional tests, the animals were sacrificed, a blood sample was taken from the left ventricle of the heart to determine the final stable nitric oxide metabolites, and the uterus section was taken for histological studies.

### 4.5. Proteinuria Estimation

Urine collection was performed for 12 h on the 42nd day from the start of the experiment, using metabolic cages (Tecniplast, Buguggiate, Italy). The amount of protein in the urine was determined by micro pyrogallol red method using the Total Protein Kit (Sigma, Ronkonkoma, NY, USA) and PE-5400B spectrophotometer (Ecopribor, Saint Petersburg, Russia) [60].

### 4.6. Determination of Stable Nitric Oxide Metabolites

We used a modification of the method for determining stable NO metabolites, which allows one-step quantitative determination of total nitrates and nitrites after deproteinization of blood serum. The principle of the method is the simultaneous reduction of nitrates to nitrites in the presence of vanadium chloride and a diazotization reaction with the subsequent development of color, the intensity of which was determined spectrophotometrically at a wavelength of 540 nm. Analysis of 100 μL of deproteinized serum was performed in 96 flat bottom well plates. The sensitivity of the method on the Labsystems Multiskan MCC 340 (Thermo Fisher Scientific, Waltham, MA, USA) is 1.7 μM. For the colorimetric determination of the nitrite ion, Griess reagent was used, consisting of equal parts of solution I (0.05% solution of N-naphthyl ethylenediamine in water) and solution II (1% solution of sulfanilamide in 30% acetic acid). Both solutions are stored in the dark at 4 °C within several months. To prepare a solution of vanadium chloride, 400 mg of VCl_3_ was dissolved in 50 mL of 1N HCl, followed by filtration through a paper filter. A freshly prepared solution was always used.

The level of NO metabolites (total concentration of nitrates and nitrites, NOx) was determined by the colorimetric method by the development of color in the diazotization reaction of sulfonamide nitrite, which is part of the Griess reagent. To construct the calibration curve, we used a 1M solution of NaNO_2_ in water, which was stored at a temperature of −20 °C; before use, it was diluted 1000 times and a series of dilutions was prepared to construct a curve [61].

### 4.7. Evaluation of Bcl-2 and BAX Expression

Placenta tissue was sampled, homogenized, and incubated for 10 min at 37 °C in the “Extract RNA” solution (Evrogen, Moscow, Russia). After lysing the sample in the reagent, it was subjected to chloroform washing, and the resulting RNA precipitate was washed with isopropanol and 70% ethanol. The concentration of the obtained RNA was measured using the spectrophotometer IMPLEN NanoPhotometer. The RNA yield was approximately 1000 ng/μL, and the concentration was equalized to 200 ng/μL.

Reverse transcription was performed using the MMLVRTSK021 set in accordance with the Protocol of the producer company (Evrogen). The mixture was carefully mixed and heated for 2 min at 70 °C to melt the secondary RNA structures and then anneal the OligoDT primer. Then, the samples were transferred to ice. The entire reaction mixture was incubated for 60 min at 40 °C in a T100™ThermalCycler (Bio-Rad, Hercules, CA, USA). To stop the reaction, the mixture was heated at 70 °C for 10 min. The resulting cDNA was diluted to a concentration of 1 ng/μL.

The level of the gene expression was evaluated relative to the values of the reference b-actin gene (Table 3). The expression at a specific point was calculated using the formula (1):

Gene expression = 2^[(Ct(Gapdh)-Ct(Target gene)].(1)

### 4.8. Histological Study

Samples of the uterus were fixed in 10% formalin, followed by paraffin filling in an STP-120 carousel machine (Microm International GMbH, City, Germany). The blocks with the standard orientation of the pieces were poured at the station for pouring biological material into EC 350 paraffin (Microm International GMBH, City, Germany). To ensure standardization, paraffin filling was carried out in the form of a multiblock of 5–6 pieces. Slices for histological examination with a thickness of 5 μm were made on a semi-automatic rotary microtome with the system of transportation and spreading of slices “NM 340E” (Microm International GMbH, Dreieich, Germany). Hematoxylin and eosin staining was carried out in a machine for staining histological sections and smears (Microm International GMbH, City, Germany). Descriptive histological studies were performed under an Axio Scope A1 microscope (Carl Zeiss Microimaging GMbH, Munich, Germany).

During the morphological study of the placenta, the density of decidual cells in the maternal part of the placenta, the density of cells in the fetal part of the placenta, and the diameter of the villi were determined. The density of decidual cells was calculated on ten sections of the maternal part in 10 non-intersecting fields of vision. A similar technique was used to determine the cell density of the fetal part of the placenta, which was determined by counting the cells that form the stroma of the villi and its shell (cytotrophoblast and symplasto-trophoblast, focusing on the nuclear elements). As the term “diameter of the villi”, we have adopted the width of the structures of the placental labyrinth, conventionally referred to as “placental villi”, the sagittal section of which was well visualized in the micropreparation.

The thickness of the maternal and fetal parts of the placenta was determined using the ImageJ 1.52u program. The thickness of the maternal part of the placenta (the thickness of the labyrinth section) was measured from well-visualized sections of the blood vessels of the umbilical cord to the border with the maternal part of the placenta (the first appearance of decidual cells). As an indicator of the thickness of the maternal part of the placenta (the spongy part), the distance from the border with the fetal part to the border of contact with smooth myocytes of the uterine myometrium was taken.

### 4.9. Statistical Analysis

For all data, descriptive statistics were used, and the data were checked for normal distribution. The distribution type was determined by using the Shapiro–Wilk test. In the case of the normal distribution, the average value (M) and standard deviation (SD) were calculated. In cases of the abnormal distribution, the median (Me) and the quartile range (QR) were calculated. In the normal distribution, the intergroup comparison was performed using one-way ANOVA and post-hoc analysis according to Tukey. In other cases, the intergroup comparison was performed using Kruskal–Wallis and Dunn’s post-hoc tests. Statistical analyses were performed using GraphPad Prism 8.0 software [62].

## Figures and Tables

**Table 1:** The ratio between the Bax and Bcl-2 expression (M ± SD, *n* = 10).

Group	Bax/Bcl-2 Ratio
Int	0.68 ± 0.1 ^y^
L-NAME	1.2 ± 0.21 ^*^
Epo (50 IU/kg)	0.83 ± 0.08 ^*,y^
pHBSP (10 μg/kg)	0.81 ± 0.08 ^*,y^
pHBSP (250 μg/kg)	0.66 ± 0.1 ^*,y^

Note: *—*p* < 0.05 in comparison with the group of intact animals; ^y^—*p* < 0.05 in comparison with the NAME group.

**Table 2:** The effect of pHBSP on the cell pool density in the fetal and maternal placenta and the villi diameter in experimental preeclampsia (M ± SD, *n* = 10 animals in each group).

Group	Decidual Cell Density, /0.008 mm^2^	Cell Dencity in the Fetal Placenta, /0.008 mm^2^	Villi Diameter, ×10^−3^μm
Int	114.2 ± 2.03 ^y^	236.9 ± 2.75 ^y^	32.18 ± 0.39 ^y^
L-NAME	22.3 ± 0.28 ^*^	78.5 ± 2.51 ^*^	16.79 ± 0.24 ^*^
Epo (50 IU/kg)	74.3 ± 0.73 ^*,y^	137.0 ± 4.17 ^*,y^	22.33 ± 0.16 ^*,y^
pHBSP (10 μg/kg)	67.7 ± 0.59 ^*,y^	92.3 ± 1.24 ^*,y^	21.01 ± 0.16 ^*,y^
pHBSP (250 μg/kg)	80.2 ± 0.75 ^*,y^	160.0 ± 3.22 ^*,y^	30.91 ± 0.17 ^*,y^

Note: * *p* < 0.05 in comparison with the group of intact animals; ^y^ *p* < 0.05 in comparison with the NAME group.

**Table 3:** Primers for evaluation of mRNA expression of target and reference genes.

Name of Primers	Nucleotide Sequence 5′- > 3′	Length
Bcl2 F	GGCCTTTTTGCTACAGGGTTTC	105
Bcl2 R	TTCTTGGTGGATGCGTCCTG	
Bax F	GTGGACAACATCGCTCTGTG	95
Bax R	AGTTCCACAAAGGCATCCCAG	
Actb F	CGCCACCAGTTCGCCAT	96
Actb R	GGGAGCATCGTCGCCC	

## Abbreviations

ADMA	Asymmetrical dimethylarginine
NOS	Nitric oxide synthase
L-NAME	N-nitro-L-arginine-methyl ether
sFlt-1	Soluble FMS-like tyrosine kinase-1
PlGF	Placental growth factor
sEng	Soluble endoglin
PAPP-A	Pregnancy-associated plasma protein-A
EPO	Erythropoietin
CED	Coefficient of endothelial dysfunction
HCl	Hydrogen chloride
NaNo2	Sodium nitrite
ADMA	Asymmetrical dimethylarginine
SBP	Systolic blood pressure
DBP	Diastolic blood pressure
NOx	Nitric oxide metabolites
qPCR	quantitative polymerase chain reaction
mRNA	messenger RNA
JAK2	Janus kinase 2
